# 3D magnetic seed localization for augmented reality in surgery

**DOI:** 10.1007/s11548-024-03066-6

**Published:** 2024-03-16

**Authors:** Pierre Ambrosini, Sara AzizianAmiri, Eliane Zeestraten, Tessa van Ginhoven, Ricardo Marroquim, Theo van Walsum

**Affiliations:** 1 Department of Surgical Oncology, Erasmus MC, University Medical Center Rotterdam, Rotterdam, The Netherlands; 2 Department of BioMechanical Engineering, Delft University of Technology, Delft, The Netherlands; 3 Department of Surgery, Amphia Ziekenhuis, Breda, The Netherlands; 4 Computer and Graphics Visualization Group, Delft University of Technology, Delft, The Netherlands; 5 Department of Radiology and Nuclear Medicine, Erasmus MC, University Medical Center Rotterdam, Rotterdam, The Netherlands

**Keywords:** 3D localization, Magnetic seed, Mixed-reality, Surgery

## Abstract

**Purpose:**

For tumor resection, surgeons need to localize the tumor. For this purpose, a magnetic seed can be inserted into the tumor by a radiologist and, during surgery, a magnetic detection probe informs the distance to the seed for localization. In this case, the surgeon still needs to mentally reconstruct the position of the tumor from the probe’s information. The purpose of this study is to develop and assess a method for 3D localization and visualization of the seed, facilitating the localization of the tumor.

**Methods:**

We propose a method for 3D localization of the magnetic seed by extending the magnetic detection probe with a tracking-based localization. We attach a position sensor (QR-code or optical marker) to the probe in order to track its 3D pose (respectively, using a head-mounted display with a camera or optical tracker). Following an acquisition protocol, the 3D probe tip and seed position are subsequently obtained by solving a system of equations based on the distances and the 3D probe poses.

**Results:**

The method was evaluated with an optical tracking system. An experimental setup using QR-code tracking (resp. using an optical marker) achieves an average of 1.6 mm (resp. 0.8 mm) 3D distance between the localized seed and the ground truth. Using a breast phantom setup, the average 3D distance is 4.7 mm with a QR-code and 2.1 mm with an optical marker.

**Conclusion:**

Tracking the magnetic detection probe allows 3D localization of a magnetic seed, which opens doors for augmented reality target visualization during surgery. Such an approach should enhance the perception of the localized region of interest during the intervention, especially for breast tumor resection where magnetic seeds can already be used in the protocol.

## Introduction

Complete tumor resection is a common surgical strategy where the entire tumor must be removed. A complete tumor resection with negative pathological margins aims to prevent additional disease burden to the patient, such as the need for a new surgery, more systemic therapies or additional radiotherapy. However, removing too much healthy tissue due to a large safety margin might increase the risk of collateral damage, organ impairment and hampered cosmetic outcomes. Therefore, it is critical to precisely localize the tumor and reduce the safety margin. In addition, surgeons often need to target tumors which are non-palpable and visually similar to healthy tissues. In such cases, providing additional information on the target position may improve the procedure and outcome. For example, in lumpectomy (breast cancer tumor resection), a seed localization system can be used [1]. Depending on the device, the technology may be based on radioactive/magnetic seed, electromagnetic (EM), radiofrequency identification (RFID) or radar reflector [2]. In any case, a radiologist first inserts the seed in the tumor using ultrasound imaging and, during surgery, the seed is tracked using a detection probe informing the distance between its tip and the seed. Nevertheless, it is still difficult to mentally reconstruct the position of the seed using only distance information. 3D localization and visualization of the seed via an augmented reality device may enhance the surgeon’s 3D perception and lead to faster and more complete tumor resection, benefiting the patient.

The purpose of our study, therefore, is to develop and assess a method to localize a magnetic seed in 3D and to demonstrate how such 3D localization could subsequently be used in an augmented reality setup to enhance the surgeon’s perception.

It has been shown that magnetic seeds contribute to improved localization in non-palpable tumor resection [3] and they have been successfully used for breast tumor surgery [4]. Recently, they have been applied for non-breast lesions [5]. Such seeds measure around 1 $\times$ 5 mm. Examples of magnetic sensing systems are Sirius Pintuition (Sirius Medical Systems B.V., The Netherlands) and Sentimag (EndoMagnetics Ltd, UK). Both systems provide a magnetic detection probe and a console unit with audible feedback. The Sirius Pintuition’s console unit displays the distance between the probe and the seed in millimeters, while Sentimag’s console displays a relative count between − 9999 and 9999. The Sirius Pintuition also allows aligning the probe above the seed with directional guidance based on visual and audio feedback.

Augmented reality in surgery has been widely studied for neurosurgery, orthopedic and spine, laparoscopic and oral surgery [6]. The proposed methods enable localization and visualization of a region of interest using image registration via video (RGB, infrared, depth-sensing, stereo, ultrasound) and, most of the time, tracking of fiducials such as optical, EM, RFID or fluorescent [7] markers. Such localization works well for rigid regions of interest and in case of minimal motion. However, it becomes much more challenging when tissue is deformed by breathing/cardiac motion, the position of the patient or the surgery itself. Deformable models have been proposed to align and keep track of the region of interest for, among others, the liver, breast, brain and gallbladder [8]. Yet, further research is necessary to achieve better robustness and error control [9].

To avoid the impact of tissue deformation, different approaches have been developed to localize and track 3D markers directly inside the body. EM tracking permits real-time sub-millimeter accurate tracking of 3D sensor poses [10]. It requires an EM field generator close to the patient and, consequently, an appropriate placement in the operating theater to not hinder surgeon’s actions. Electro-surgical knives or ferro-magnetic tools in the vicinity of the field generator may reduce the accuracy of the localization. More importantly, although sensors can be very small (diameter less than 1 mm), they are typically not wireless, imposing a serious limitation for surgery applications such as tumor resection. Nonetheless, augmented reality methods with wired sensors have been proposed for rectal cancer [11] or using tracked needles and an ultrasound probe for breast-conserving surgery [12].

The Calypso 4D Localization System (Varian Medical Systems, USA) uses passive transponders inside a magnetic field. Each transponder has dimensions of 1.85x8 mm, and the real-time 3D tracking reaches sub-millimeter accuracy [13]. This technology is used in radiotherapy, e.g., for prostate cancer [14]. Recently, studies have been carried out for image guidance with tumor tracking during surgery [15]. By tracking two or three transponders [16], rigid alignment with a preoperative 3D scan can be obtained. Tracking via Calypso has been shown to be accurate in an operating room, but positioning the EM array close to the patient table can reduce the tracking accuracy [17]. Furthermore, one must ensure that the EM array position is close to the patient’s region of interest and does not obstruct the surgeons during the intervention.

The contributions of this paper are twofold. First, we present and evaluate a method to localize a magnetic seed in 3D by tracking the detection probe. The tracking uses QR-code detection with a head-mounted display (HMD) or optical tracking with retroreflective spheres. Second, a proof-of-concept is demonstrated with a breast phantom and an augmented reality overlay with the HMD.

**Fig. 1:**
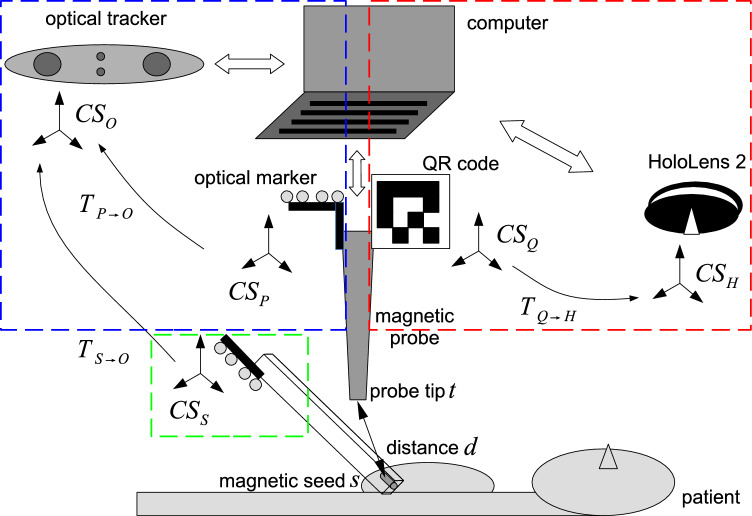
3D magnetic seed tracking setup using a magnetic detection probe and an optical tracker (blue box) or an HMD (red box). The seed holder (green box) is used only as a ground truth seed for the evaluation of the probe tip calibration and the seed localization experiment

## Materials and methods

Our setup for 3D localization of a magnetic seed uses a commercially available magnetic detection system coupled with either an HMD with a camera sensor or an optical tracking system (Fig. 1). Next, the common setup is described, followed by a description of each system (magnetic sensing system, QR-code and HMD, and optical tracking).

### Setup

The magnetic detection system tracks the magnetic seed using a probe. The system provides the distance between the seed and the magnetic detection probe tip. For tracking the 3D pose of the magnetic sensing probe, a QR-code or optical marker is attached to the probe. These markers permit tracking the probe with a camera from an HMD or an optical tracker, respectively.

This setup can be used to acquire pairs of probe poses and distances between the seed and the tip while moving the probe around. With a sufficient number of pairs, the 3D position of the seed in the coordinate system of the HMD or optical tracker can be determined.

In the following part, we mathematically formulate the localization for the QR-code marker and HMD. The formulation for the optical tracking method is similar and details are in Section “Optical tracking”.

At any time point *i* during acquisition, the magnetic detection probe provides the distance *d*(*i*) between the seed and the probe’s tip. Assuming the two following coordinate systems (CS):

- CS_H_ HMD CS, i.e., the CS of the 3D position tracker, and
- CS_Q_ marker CS (centered on the tracked marker),

we define two fixed unknown positions in homogeneous coordinates, one for the seed position and one for the tip of the probe:

- $s^H = [s^H_x, s^H_y, s^H_z, 1]^\intercal$, the seed position in CS_H_;
- $t^Q = [t^Q_x, t^Q_y, t^Q_z, 1]^\intercal$, the tip of the probe in CS_Q_.

The affine matrix for transforming coordinates from CS_Q_ to CS_H_ at time point *i* is:

$$\begin{aligned}T_{Q \rightarrow H}(i) = \begin{bmatrix} m_{00}(i) &{} m_{01}(i) &{} m_{02}(i) &{} m_{03}(i)\\ m_{10}(i) &{} m_{11}(i) &{} m_{12}(i) &{} m_{13}(i)\\ m_{20}(i) &{} m_{21}(i) &{} m_{22}(i) &{} m_{23}(i)\\ 0 &{} 0 &{} 0 &{} 1\\ \end{bmatrix} \end{aligned}$$

and can be obtained from the QR-code tracking setup (see Section “QR-code and head-mounted display”).

As the QR-code is rigidly attached to the probe, the tip of the probe in CS_H_ at time point *i* can then be computed as follows:

$$\begin{aligned} t^H(i) = T_{Q \rightarrow H}(i) \cdot t^Q \quad , \end{aligned}$$

and thus:

$$\begin{aligned} t^H_x(i)= & {} t^Q_x m_{00}(i) + t^Q_y m_{01}(i) + t^Q_z m_{02}(i) + m_{03}(i) \quad , \\ t^H_y(i)= & {} t^Q_x m_{10}(i) + t^Q_y m_{11}(i) + t^Q_z m_{12}(i) + m_{13}(i) \quad , \\ t^H_z(i)= & {} t^Q_x m_{20}(i) + t^Q_y m_{21}(i) + t^Q_z m_{22}(i) + m_{23}(i) \quad . \end{aligned}$$

Using the fact that, at time point *i*, the distance between the tip and the seed *d*(*i*) is known (obtained from the magnetic seed system), we have the following squared Euclidean distance expression for the probe tip position, $t^H(i)$, and the seed, $s^H$, both in HMD coordinates:

$$\begin{aligned} ||s^H - t^H(i)||^2 = d(i)^2 \quad , \end{aligned}$$

which implies

$$\begin{aligned} (s^H_x - t^H_x(i))^2 + (s^H_y - t^H_y(i))^2 + (s^H_z - t^H_z(i))^2 = d(i)^2 \quad , \end{aligned}$$

and thus:

1 $$\begin{aligned}&(s^H_x - t^Q_x m_{00}(i) - t^Q_y m_{01}(i) - t^Q_z m_{02}(i) - m_{03}(i))^2 \nonumber \\&\quad + (s^H_y - t^Q_x m_{10}(i) - t^Q_y m_{11}(i) - t^Q_z m_{12}(i) - m_{13}(i))^2\nonumber \\&\quad + (s^H_z - t^Q_x m_{20}(i) - t^Q_y m_{21}(i) - t^Q_z m_{22}(i) - m_{23}(i))^2 \nonumber \\&\quad = d(i)^2 \quad , \end{aligned}$$

with unknowns $s^H_x, s^H_y, s^H_z, t^Q_x, t^Q_y$ and $t^Q_z$.

Each time point *i* yields an equation, and the resulting system of equations can be numerically solved using a least square fitting technique. In theory, six different poses are sufficient, but in practice, due to acquisition inaccuracies (seed/tip distance error, QR-code detection error, temporal synchronization error), more time points / poses are needed to solve this set of equations reliably.

The tip position $t^Q$ is fixed over time as the QR-code is rigidly linked with the probe geometry. Consequently, we can perform a probe tip calibration beforehand once and then, for the seed localization, substitute the calibrated tip values $t^Q_x, t^Q_y$ and $t^Q_z$ in Eq. 1, reducing the number of unknowns to three: $s^H_x$, $s^H_y$ and $s^H_z$. During the least square fitting, $s^H$ and $t^Q$ are initialized to [0, 0, 0, 1] for the probe tip calibration. For the seed localization, $t^Q$ is known (and so $t^H(i)$ is as well). We can then initialize the fitting with $s^H$ to $[t^H_x(i), t^H_y(i), t^H_z(i), 1]$ with *i* being the time point when *d*(*i*) is smallest, i.e., when the probe tip is closest to the seed. This is to ensure a fast convergence.

### Magnetic sensing system

We used the magnetic detection system Sirius Pintuition (Sirius Medical Systems B.V., The Netherlands). A research version of the software allowed us to connect to the probe via USB and record for each timestamp a distance between the probe tip and the seed (in millimeters) with a frequency of 45 Hz. When the probe is too far away from the seed no distance is given. The probe has a maximum detection range of 50 mm.

**Fig. 2:**
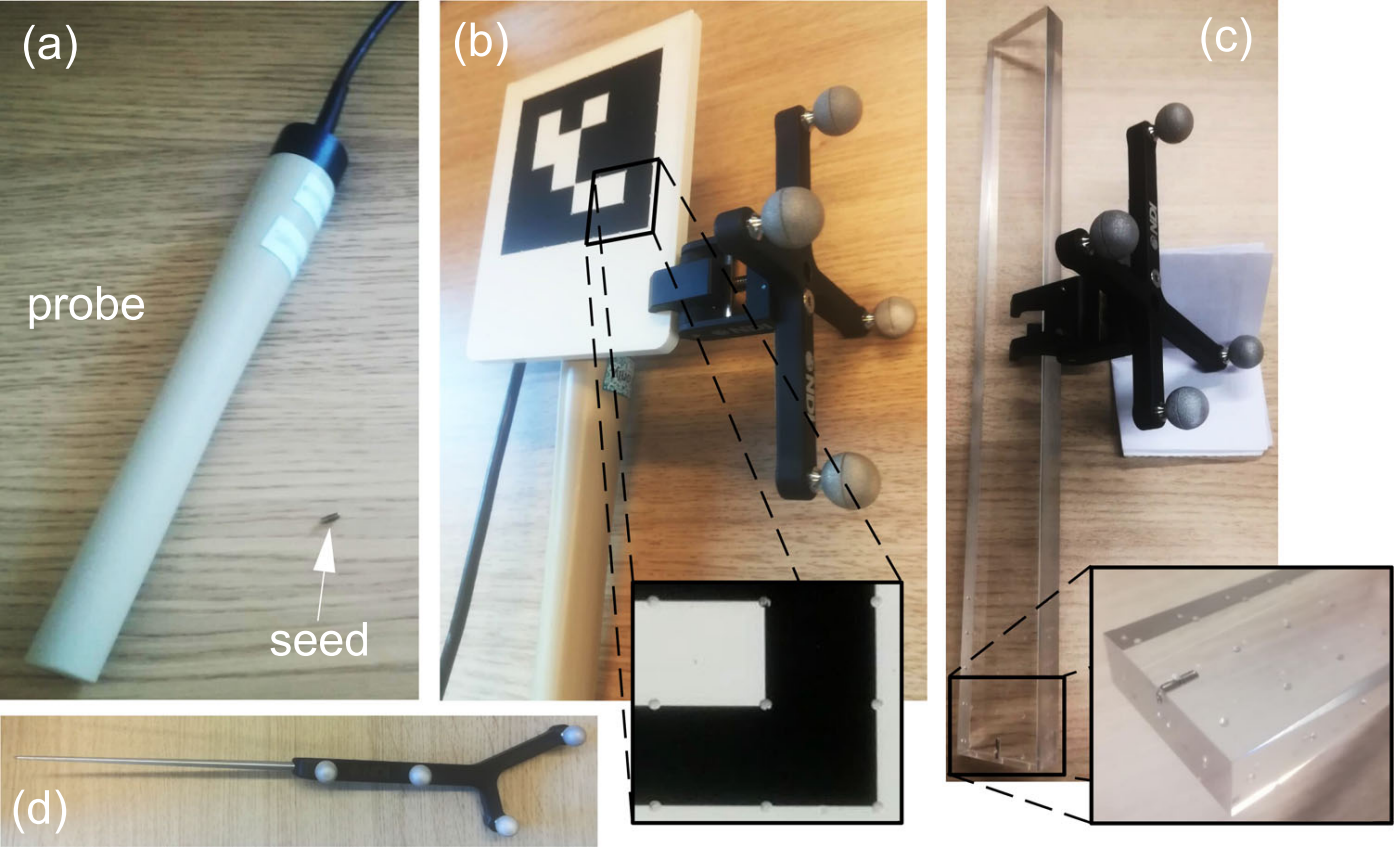
**a** Sirius Pintuition probe and magnetic seed. **b** QR-code and optical marker attached to the probe. The QR-code has divots to be localized by a pointer. **c** Seed holder: magnetic seed in a rigid plastic plank with the optical marker attached and divots. **d** Optical pointer used to register the QR-code and seed holder with regard to the optical tracker CS_O_

### QR-code and head-mounted display

The HMD used is a HoloLens 2 (HL2), Microsoft, USA. The integrated RGB camera sensor acquires images with a resolution of 1280 $\times$ 920 pixels at around 15 Hz. A 6x6 cm QR-code is attached at the distal end of the probe (Fig. 2b). The AruCo library is used for the detection of the QR-code in the camera images. At time *i*, when the QR-code is detected in the camera image, we obtain the transformation matrix $T_{Q \rightarrow C}(i)$ going from CS_Q_ to the camera CS_C_. By virtue of its integrated simultaneous localization and mapping (SLAM) algorithm, the HL2 keeps CS_H_ fixed in the world/reality space, regardless of its motions and pose. Therefore, the transformation matrix $T_{C \rightarrow H}(i)$ going from CS_C_ to CS_H_, provided by the HL2, can be used to determine $T_{Q \rightarrow H}(i)$. Together with the distance at time point *i* from the magnetic sensing system, this provides all input required for Eq. 1.

A prototype mixed-reality application was developed to visualize the seed position on the patient using the HL2. After computing the 3D seed position $s^H$, a 3D model (three orthogonal axes intersecting at $s^H$, see Fig. 7) is displayed at the corresponding position. With the HL2, the user can look at the target from various directions to have a better perception of the seed’s position.

### Optical tracking

The optical tracker used is a Polaris Vega (Northern Digital, Canada). The probe is tracked using an optical marker with four passive reflective spheres (Fig. 2b). The marker attached to the probe gives at time *i* the transformation matrix $T_{P \rightarrow O}(i)$ (see Fig. 1). We replace the HMD coordinate system CS_H_ and the marker coordinate system CS_Q_ with the optical tracker CS_O_ and optical marker CS_P_ when solving the equations.

## Protocols and calibrations

In order to perform the 3D magnetic seed localization during surgery, temporal and probe tip calibration needs to be done beforehand, and an acquisition protocol needs to be followed to maximize the accuracy of the probe tip calibration and the seed localization.

### Temporal calibration

Calibration is performed to temporally synchronize the HL2 (resp. the optical tracker) and the computer connected to the magnetic probe. At the beginning of each experiment, the magnetic probe is moved slowly up and down close to the magnetic seed for about 10 s. The distances *d*(*i*), the up-axis translations of the QR-code marker pose $T_{Q \rightarrow H}(i)$ (resp. the optical marker pose $T_{P \rightarrow O}(i)$) are acquired, normalized and plotted over time. The two curves are manually aligned by inserting a time offset. These offsets will be used to synchronize the subsequent acquisition in the experiment.

**Fig. 3:**
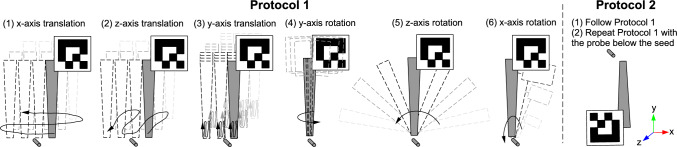
Acquisition protocols to follow to get discriminating and distributed probe poses. Protocol 2 is used during tip calibration, where the probe can be moved under the seed

### Acquisition protocol

Solving Eq. 1 requires an acquisition with discriminating and well-distributed probe poses; without spatial variation, there is insufficient information to solve the equation accurately. Therefore, the probe motion protocols are designed to cover a wide range of poses in order to obtain an optimal seed localization. Figure 3 summarizes the acquisition protocols. Protocol 1, used for seed localization, consists of moving the probe slowly around the seed with translations and rotations around all the axes. Protocol 2 is used for the tip calibration. As there is no patient in this case, the probe can be moved below the seed. The same motion of Protocol 1 is applied twice: once above the seed and one vertically flipped below the seed. Thus, Protocol 2 has more distributed probe poses around the seed than Protocol 1 and is expected to be more robust to acquisition errors (seed/tip distance error, optical tracking error and temporal synchronization error).

**Fig. 4:**
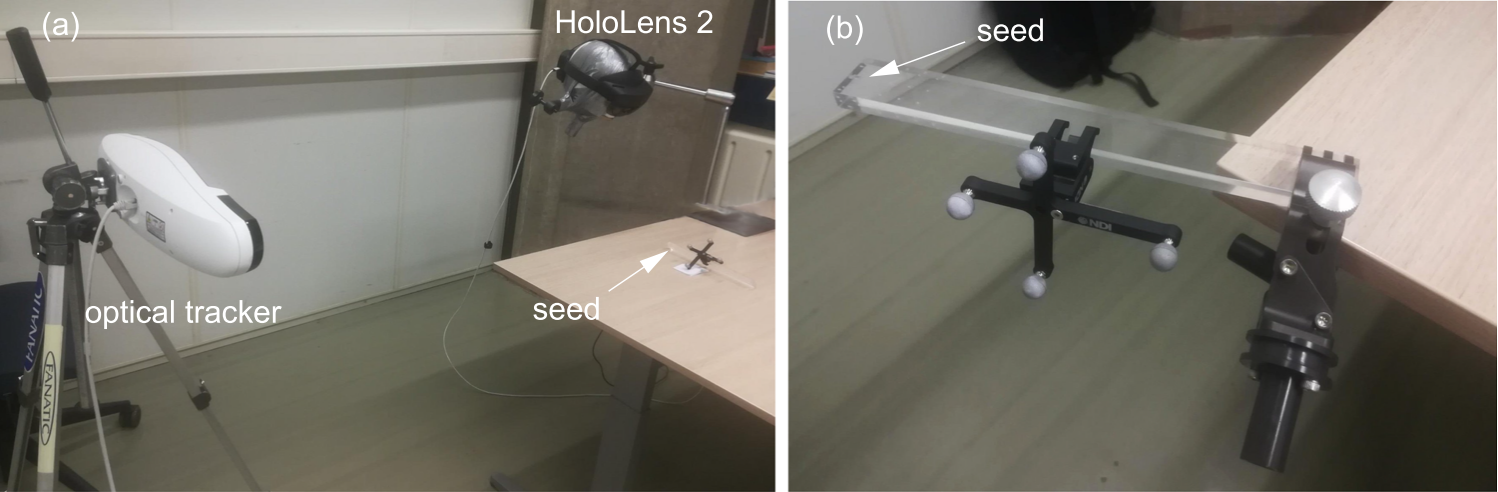
**a** 3D seed localization evaluation setup with HoloLens 2 and optical tracker. **b** Probe tip calibration setup. The probe can be manipulated around the seed with ease

### Probe tip calibration

Probe tip *t* is computed using the optical tracking setup. During the acquisition (Fig. 4b), we follow Protocol 2 as depicted in Fig. 3. This gives, for every time *i*, the probe pose and seed/tip distance: ($T_{P \rightarrow O}(i)$, *d*(*i*)). Equation 1 is solved to obtain $t^P$ and $s^O$, while $t^Q$ is computed using $T_{P \rightarrow Q}$.

## Experiments

Before describing the seed localization experiment and the breast phantom experiment, we explain how the ground truth is acquired and computed for the evaluation (optically tracked rigid seed holder, correspondences between the HMD and the optical tracking via the QR-code).

### Reference standard

We track the seed position to serve as the reference. A rigid holder was constructed where the magnetic seed was inserted. It also contains divots of which the positions are known with regard to the seed (Fig. 2c). An optical marker is attached to the seed holder, giving the transformation matrix $T_{S \rightarrow O}(i)$ (see Fig. 1). Using an optically tracked pointer, the position of 18 divots was determined in CS_O_ and then in CS_S_ using $T_{O \rightarrow S}(i)$. As all divot positions are known with regard to the seed, the ground truth seed position $s_\textrm{gt}^S$ in CS_S_ was estimated using point-based registration (PBR) and then $s_\textrm{gt}^O$ was computed using $T_{S \rightarrow O}(i)$. During the probe tip calibrations and the seed localization experiment, the rigid holder is static and nothing touches it in order to minimize errors in the ground truth.

Correspondences between the QR-code and the optical marker attached to the probe are necessary to evaluate the QR-code and HMD setup using optical tracking as ground truth. Therefore, the position of nine divots on the QR-code was acquired, and PBR was performed to obtain $T_{P \rightarrow Q}$. For every experiment, in order to compensate for the QR-code detection errors, the spatial correspondence between the HMD space CS_H_ and the optical tracker space CS_O_ is averaged over all the probe poses and computed at the end of the experiment. At time *i*, we know $T_{Q \rightarrow H}(i)$ and $T_{P \rightarrow O}(i)$ and so $T_{H \rightarrow O}(i)$ (using $T_{P \rightarrow Q}$). $T_{H \rightarrow O}$ is computed as the composition of the mean translation and the chordal L2 mean rotation of all $T_{H \rightarrow O}(i)$ transformations [18].

In order to evaluate how close every single spatial correspondence $T_{H \rightarrow O}(i)$ is to the mean transformation $T_{H \rightarrow O}$, we compute the Euclidean distance *D* at each time *i* between the QR-code center position computed using the camera tracking ($T_{Q \rightarrow H}(i)$) and using the optical tracking ($T_{Q \rightarrow O}(i)$; considered here as the ground truth):

2 $$\begin{aligned} D = || T_{H \rightarrow O}T_{Q \rightarrow H}(i)[0,0,0,1]^\intercal - T_{Q \rightarrow O}(i)[0,0,0,1]^\intercal || \nonumber \\ \end{aligned}$$

Distance *D* can be used as a metric to evaluate the QR-code localization accuracy by the camera sensor ($T_{Q \rightarrow H}(i)$). At time *i*, a large Euclidean distance in Eq. 2 shows potentially a QR-code detection outlier, deviating from the mean spatial correspondence $T_{H \rightarrow O}$.

### Seed localization experiment

We evaluate the seed localization for both the QR-code/HMD setup and the optical tracking setup. The probe tip position *t* is known from the probe tip calibration (Section “Probe tip calibration”). To prevent any impacts from the HL2’s SLAM motion tracking, the HL2 is fixed and attached to a mounting arm (Fig. 4a).

The acquisition for seed localization follows Protocol 1 (Fig. 3), mimicking a clinical situation where the probe cannot be used under the body. This experiment has been performed twice for approximately 2 min each time. The Euclidean distance between the computed seed $s^H$/$s^O$ and the tracked seed $s_\textrm{gt}^O$ are reported.

**Fig. 5:**
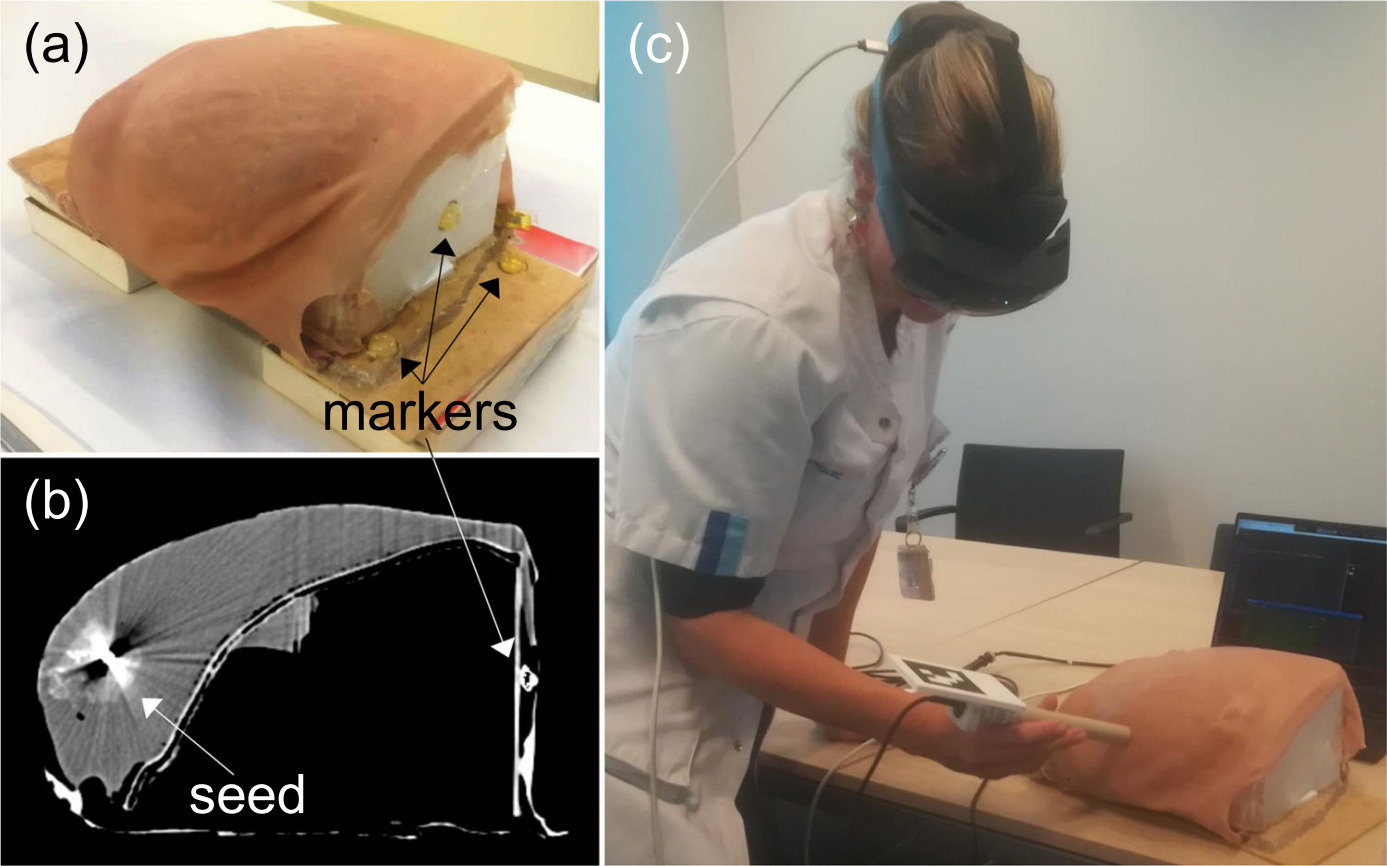
**a** Breast phantom with a magnetic seed inside and markers outside. **b** Computed tomography slice of the breast phantom. **c** Seed localization demo with a surgeon

### Breast phantom experiment

A breast phantom (Fig. 5a) where a magnetic seed was inserted was constructed for assessment of the method. The seed is not visible from the outside, and the tissue (using soft polyvinyl chloride plastisol) mimics the properties of breast tissue when touched. Here, compared to the previous experiment, the rigid seed holder cannot be used. So, ten fiducial markers (PinPoint for Image Registration, Beekley Medical, USA) that can be localized by an optical pointer were attached to the rigid part of the phantom (thorax and support). A 3D computed tomography (CT) scan of the breast phantom was acquired where the magnetic seed and the markers are visible (Fig. 5b). The CT resolution is 957 $\times$ 512 $\times$ 512 with a voxel size of 0.6 $\times$ 0.6 $\times$ 1.0 mm. By localizing the fiducial markers and using PBR, the CT scan was aligned with the phantom in the optical tracker space CS_O_, and the seed position $s_\textrm{ct}^O$ from the CT scan is then known in CS_O_ as well. The correspondence between the HL2 space CS_H_ and the seed $s_\textrm{ct}^O$ is made via the optically tracked QR-code as explained in Section “Reference standard”.

For this experiment, the HL2 is not fixed and is worn by the person manipulating the magnetic probe. Visual feedback is displayed when the QR-code is detected with a purple square around it. When the calibration is done, a visual target is shown at the position of the computed seed (see Section“QR-code and head-mounted display”). Due to the HL2’s integrated SLAM, the visual target stays at the same place regardless of the pose of the person wearing the HL2.

The acquisition follows Protocol 1 (Fig. 3) and lasts 2 min. The Euclidean distance between the computed seed $s^H$/$s^O$ and the tracked seed $s_\textrm{ct}^O$ is reported. Three users participated in the experiment. User 1 is a researcher familiar with the HL2 and had already performed the calibration task several times. User 2 is a student with a technical and clinical background. User 3 is a researcher with experience in mixed-reality. Users 2 & 3 had never manipulated a magnetic detection probe before. Users 1 and 3 performed the experiment two times each. Due to time schedule constraints, User 2 performed the experiment only once after training. In order to minimize spatial projection errors, prior to using the application, the HL2 was individually calibrated to each user using the HL2’s eye calibration procedure.

**Table 1:** Probe tip calibrations done before the seed localization and the breast phantom experiments, using only optical tracking as ground truth

Experiment	Protocol	Duration in s	Poses number	Error in mm
Example	1	138	2523	4.09
Seed localization	2	280	5144	0.69
Breast phantom	2	312	5232	1.79

The first tip calibration was only done to demonstrate the difference between Protocol 1 and 2. Error is the Euclidean distance between the computed seed and the ground truth seed position

**Fig. 6:**
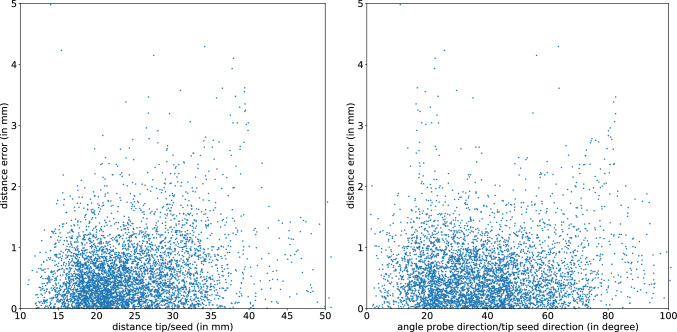
Spatial distribution of the distance errors between the probe tip and the seed given by the magnetic sensing system. The left Figure shows the errors in function of the distance of the probe tip with regard to the seed. The right Figure shows the errors in function of the direction of the probe with regard to the axis tip-seed. An angle of $0^{\circ }$ indicates that the seed is in front of the probe. The probe poses and distance errors come from the probe tip calibration done before the seed localization experiment

**Table 2:** Experiments result using Protocol 1 and after a probe tip calibration

Experiment	Duration in s	Optical Poses number	Error in mm	QR-code Poses number	Error in mm	*D* in mm median/max
*Seed localization*						
First test	129	2300	0.59	800	1.02	2.97 / 22.17
Second test	138	2383	1.11	884	2.13	5.00 / 32.49
Average			0.8		1.6	
*Breast phantom*						
User 1- First test	121	1771	1.84	549	9.43	4.22 / 100.39
User 1 - Second test	120	1921	1.43	659	0.88	1.96 / 22.77
User 2 - First test	121	1812	1.90	635	2.92	2.75 / 30.97
User 3 - First test	127	1660	2.51	515	7.03	3.83 / 31.84
User 3 - Second test	130	1632	2.65	667	3.18	2.6 / 38.88
Average			2.1		4.7	

Error is the Euclidean distance between the computed seed and the ground truth seed position. *D* is the QR-code detection error metric

**Table 3:** Fiducial registration error (in mm) of the point-based registrations done before the experiments

	Seed localization			Breast phantom	
Point-based registration	QR-code divots	Seed holder divots	QR-code divots	Seed holder divots	Breast phantom markers^∗^
Fiducial registration error (RMSE)	0.07	0.29	0.12	0.21	[1.16, 3.39]
Maximum error	0.16	0.93	0.28	0.59	[3.38, 3.99]

Note that the seed holder for the breast phantom experiment is used only during the probe tip calibration

^∗^Interval among the 5 user experiments

## Results

During the probe tip calibration, the seed was optically tracked via the seed holder. So, as an evaluation, the Euclidean distance between the computed seed $s^O$ and the ground truth tracked seed $s_\textrm{gt}^O$ can be determined. Table 1 shows these results for the probe tip calibrations done before both the seed localization and the breast phantom experiments. We added a dummy probe tip calibration using only Protocol 1 to demonstrate the need for Protocol 2 (more diverse probe poses covering all degrees of freedom around the seed).

With the assumption that the optical tracking acquisition and the temporal calibration between the optical and magnetic probe have no errors (which is in reality not possible), the errors by the magnetic sensing system can be computed by comparing the distance probe tip/seed given by the magnetic system and the distance calibrated probe tip/ground truth seed. Those distance errors are displayed in Fig. 6 for the probe tip calibration done before the seed localization experiment. The left Figure shows the errors in function of the distance of the probe tip with regard to the seed. The right Figure shows the errors in function of the direction of the probe with regard to the axis tip-seed. Most distance errors are below 1 mm and no clear correlation can be made with the outliers in function of the orientation of the probe or its proximity to the seed. The magnetic sensing system seems quite robust in this probe range and orientation.

Table 2 summarizes for each experiment the acquisition time, number of acquired probe poses, Euclidean distance between the computed seed and the ground truth, and the median/max of the QR-code detection error metric *D*. Additionally, we note that the use of a calibrated probe tip is crucial to improve the accuracy of the localization with Protocol 1 (e.g., with the QR-code/HMD setup, the distance is reduced from 8.83 to 1.02 mm in the first test of the seed localization experiment).

Table 3 shows the fiducial registration error (RMSE) of the PBRs performed to compute $T_{P \rightarrow Q}$, $s_\textrm{gt}^O$ and $s_\textrm{ct}^O$.

**Fig. 7:**
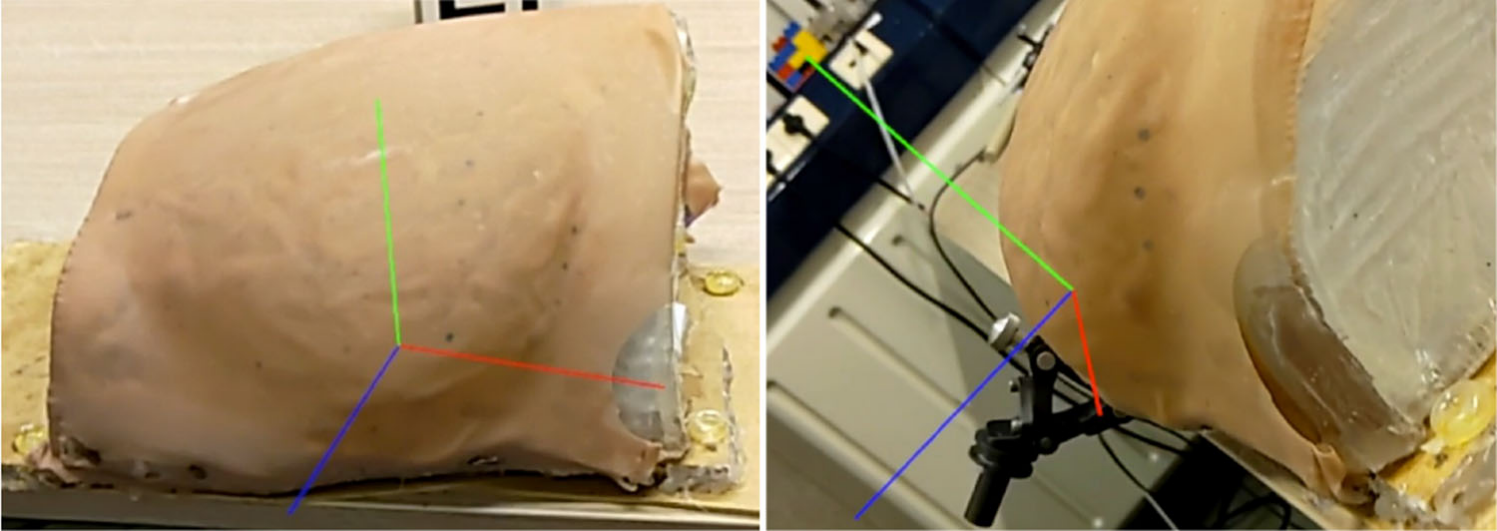
Two captures from the HL2 point of view with its 3D holographic overlay. The position of the computed seed is visualized as the intersection of the three red, blue, and green orthogonal segments. The position and depth are poorly perceived in the static images, but become more clear when wearing the HMD

In the breast phantom experiment, the computed seed position $s^H$ is overlaid using the HL2 (Fig. 7).

## Discussion

We proposed a method to localize and visualize in 3D the position of a magnetic seed using a detection probe and HL2 device with QR-code or optical tracking. With an acquisition following Protocol 1, the average Euclidean distance between the 3D computed seed and the ground truth is 1.6 mm using the QR-code and 0.8 mm using optical tracking. This is sufficient for surgical applications where seed localization and visualization are needed. In lumpectomy, the magnetic seed is already used in some hospitals. The proposed augmented reality method could then be easily integrated into the current protocol and help the surgeons gain a better 3D perception of where the magnetic seed and the tumor are. Following the procedure, a choice should be made between using the less accurate QR-code or the more cumbersome optical tracking setup.

Seed localization using subsets of the acquisition shows that shorter acquisition time results in more accuracy variation. Protocol 1 needs to be completely followed to add robustness to the method. Consequently, a two-minute acquisition does not permit live tracking or following a moving seed.

The optical tracking setup is more accurate than the HMD with QR-code setup, since the largest measurement error is due to the QR-code pose estimation and detection. The metric *D* (acting as a QR-code localization accuracy metric) has a median Euclidean distance between 2 and 5 mm but can have outliers with a Euclidean distance up to 100.39 mm (Table 2). Such outliers happen when the QR-code is far from the camera sensor and not aligned with the camera projection plane. Similarly, outliers with a large rotational error in the pose estimation could be addressed. Overall, detecting and removing the QR-code pose estimation outliers should potentially improve the computed seed position accuracy. Moreover, to reduce pose estimation errors, a higher camera resolution could be used at the expense of slower framerates. Finally, the intrinsic and distortion parameters of the camera sensor should be carefully calibrated to improve accuracy. Following Gsaxner et al. [19], the HL2’s factory camera parameters should be avoided, and camera calibration should be performed instead.

With the breast phantom experiment, the computed seed position is less accurate than the seed localization evaluation using the seed holder. The average distance between the computed seed and the ground truth is 4.7 mm using the QR-code and 2.1 mm using optical tracking. The combination of three factors is a possible explanation for the lower accuracy. First, the ground truth is less reliable than the tracked seed holder because the seed and markers position in the breast CT scan is less accurate due to the scan resolution. Second, during the acquisition, the seed is not visible to the user, and for some orientations, the probe has to push the breast tissue to be in the 50 mm detection range. It is consequently more difficult to follow Protocol 1 and the acquired poses are less evenly distributed. Here, user experience seems to be a key factor in improving accuracy. Augmented visual aids could be given in order to improve probe manipulation. Lastly, solely with the HMD using QR-code setup, the HL2 is not fixed and follows the user’s head motion. Errors in the HL2 integrated SLAM to keep the coordinate system CS_H_ fixed have to be considered. A study on HL2 spatial mapping accuracy should be conducted. Using a fixed QR-code close to the patient as a coordinate reference system could be a solution [20]. That would add, however, another constraint to always have the QR-code in sight of the HL2 camera.

Our mixed-reality application to visualize the seed was straightforward and may benefit from further improvements and evaluation. In order to give the most accurate visualization for the surgeons, several directions for future experiments with the HL2 are foreseen. First, we should ensure a hologram’s stability with correct device settings such as reprojection [21]. Second, depth perception associated with visualization, especially with HMDs, is a challenging topic and should be tackled [22]. For breast-conserving surgery, seed localization could be combined with 3D scan overlay methods [23]. The position of the tumor regarding the seed and the artifacts caused by the seed in the 3D scan are challenges that should be addressed (e.g., using a non-rigid registration with a previous scan where the seed has not been yet inserted). In the end, better tumor localization and visualization should help reduce the margin during resection and improve patient outcomes.

## Conclusion

We propose a 3D magnetic seed localization method and achieved an average location error of up to 4.7 mm using QR-code and 2.1 mm with optical tracking. This could help surgeons to better understand the operating scene using augmented reality, especially for lumpectomies where magnetic seeds can already be used in the protocol.
